# Immunomodulatory Agents for Multiple Myeloma

**DOI:** 10.3390/cancers14235759

**Published:** 2022-11-23

**Authors:** Jiří Minařík, Sabina Ševčíková

**Affiliations:** 1 Department of Hemato-Oncology, Faculty of Medicine and Dentistry, Palacky University Olomouc and University Hospital Olomouc, 779 00 Olomouc, Czech Republic; 2 Babak Myeloma Group, Department of Pathological Physiology, Faculty of Medicine, Masaryk University, 625 00 Brno, Czech Republic

## Introduction

The treatment of multiple myeloma (MM) has undergone a significant paradigm shift in the last 20 years, from conventional chemotherapy to more tumor-specific treatments, based on the interference with pathogenesis of the malignant clone as well as the bone microenvironment. Indeed, the introduction of novel drugs with “biological mechanisms of action” into MM treatment has established a worldwide trend towards chemotherapy-free regimens with lower toxicity (or significantly different toxic profiles) and targeted mechanisms of action. Immunomodulatory drugs (IMiDs) represent the cornerstone of the evolution of novel therapy in MM.

## 1. Thalidomide

The first immunomodulatory drug used for MM was thalidomide. Despite its controversial history, thalidomide showed promising outcomes in relapsed and refractory MM in the late 1990s. Additionally, very soon, it became a part of standard regimens in all phases of MM, including the first-line setting for both transplant-eligible and -ineligible patients, in maintenance after stem cell transplant and of course in relapsed settings of MM [1,2,3,4,5,6,7,8,9]. The MPT regimen (melphalan, prednisone, and thalidomide) became the new golden standard after more than 30 years of the MP (melphalan and prednisone) doublet in transplant-ineligible patients [6]. Novel regimens with thalidomide substituted the dominant role of VAD induction (vincristine, doxorubicin, and dexamethasone) in the majority of transplant-eligible patients [10,11,12,13,14].

The major issue of thalidomide-based therapy was toxicity, including teratogenicity, thromboembolic events, rash, constipation and especially neurotoxicity [15]. Whereas the former toxicities were preventable and manageable, neurotoxicity remained an issue as it caused both sensitivity and motor impairment and was not completely reversible, even after stopping the drug. On the other hand, unlike the majority of other drugs used in MM therapy, hematological toxicity was very rare, making it a useful drug in cytopenic patients [16].

## 2. IMiDs—Mechanism of Action

Together with attempts to optimize thalidomide-based therapy and reduce long-lasting neuropathy (and possibly teratogenicity), research was carried out in order to reveal the mechanism of action of thalidomide and to synthesize derivates with higher efficacy and lower toxicity. The mechanism of action was initially not fully understood due to its limited in vitro activity. The first reports described thalidomide as having a dual mechanism of action—direct tumor cytotoxicity and indirect interference in the bone marrow microenvironment [2,17]. The known mechanisms included the downregulation of NF-κB signaling, thus inhibiting proliferation and promoting apoptosis, or the induction of c-jun terminal kinase (JNK)-dependent caspase-8-mediated apoptosis [18]. The indirect mechanisms include the TNF-α-induced density of the adhesion molecules ICAM-1 and VCAM-1, as well as the inhibition of IL-6 and vascular endothelial growth factor (VEGF), thus disabling tumor growth and proliferation. Interference with IGF-1 and FGF genes, which are responsible for the activation of integrins and the impairment of AKT signaling, is responsible for the anti-angiogenic effects of thalidomide [19,20]. A very complex mechanism covers the immunomodulatory role of thalidomide via the regulation of the TNF-α-, IL-6-, IL-1β-, IL-12- and CD28-mediated stimulation of CD4+ and CD8+ T-cells.

Soon after the introduction of thalidomide into routine practice, novel IMiDs were developed with similar structures. These were lenalidomide and later pomalidomide. Lenalidomide was first introduced in 2004 by the Celgene company as CC-5013. Unlike thalidomide, lenalidomide does not need metabolic activation, which enabled deeper insights into the IMiDs’ mechanism of action [16]. Most of the effects (both positive and negative), including teratogenic toxicities, were found to be caused by the IMiD-mediated inactivation of cereblon and the degradation of its transcription factors, Ikaros and Aiolos [21,22]. Thalidomide and its derivatives bind to cereblon (lenalidomide and pomalidomide bind more strongly than thalidomide), which leads to the downregulation of IRF4. IRF is a survival factor in MM and its inhibition leads to anti-proliferative effects caused by the induction of the degradation of two essential transcription factors—Ikaros (Ikaros family zinc finger protein 1) and Aiolos (Ikaros family zinc finger protein 3) [23]. The most important pathogenetic findings suggest that the substrate recognition by cereblon depends on the structure of the ligands that bind to cereblon [23]. The modification of the cereblon substrate’s specificity is, however, also the reason behind the teratogenicity of IMiDs [23].

Teratogenicity was the reason for a limited approval of thalidomide in only a few defined conditions, and it is the reason for strict pregnancy-prevention programs in IMiD-based therapies. Recent research identified possible neosubstrates responsible for IMiDs’ teratogenicity, such as promyelocytic leukaemia zinc finger (PLZF)/ZBTB16 protein; still, the mechanism seems to be more complex and probably inseparable from IMiD anti-tumor activity [24].

## 3. Lenalidomide

Lenalidomide, the first thalidomide derivative, soon found its largest utility in MM. The initial clinical trials revealed feasibility in the relapsed setting. Still, there were limitations due to its myelosuppressive toxicity, which was not observed in thalidomide trials. In combination with other myelosuppressive drugs (melphalan, cyclophosphamide, and anthracyclines), hematological toxicity seemed to be an issue, though it was manageable [25]. Nevertheless, the therapeutic outcomes were impressive, especially in patients who were able to sustain treatment for a long time, especially as the clinical trials started to be designed “until progression”. The overall response rates (ORR) of RD combination (lenalidomide and dexamethasone) in relapsed multiple myeloma exceeded 60%, with a median progression-free survival (mPFS) of over 11 months [26,27]. The combination enabled lower doses of both drugs with limited toxicity and set up a new gold standard for both relapsed settings and newly diagnosed MM [26,27,28,29,30]. At the moment, RD-based regimens are some of the most effective regimens in relapsed and/or refractory MM when combined with proteasome inhibitors—bortezomib (VRD), carfilzomib (KRD), ixazomib (IRD)—or with monoclonal antibodies—elotuzumab (EloRD) and daratumumab (DRD)—with a consistent ORR over 70% and mPFS over 20 months, which was previously only seen in newly diagnosed patients with standard-risk MM [31,32,33,34,35].

Some of these combinations have been tested in frontline settings and are very likely to become the new preferred option for transplant-ineligible patients (especially DRD regimen) [36]. In transplant-eligible patients, lenalidomide is becoming the cornerstone of induction therapy and maintenance treatment. Lenalidomide maintenance after autologous stem cell transplant added 2 years to the progression-free survival and 2.5 years to overall survival of MM patients, thus shifting the prognosis of MM even further [37].

## 4. Pomalidomide

Pomalidomide is the “youngest” IMiD routinely used in clinical practice. It is considered the most efficient drug among the IMiDs; however, it is generally used in patients with advanced MM after pretreatment with several drugs, including lenalidomide [38,39]. This might affect the final outcomes despite the evidence of only the minimal cross-resistance of these drugs. Pomalidomide is metabolized predominantly by the liver (unlike lenalidomide) and does not need dose reductions in patients with renal failure [38]. Similarly to lenalidomide, the major toxicities of pomalidomide include neutropenia, anemia and thrombocytopenia, with the most common non-hematologic adverse events being infection (pneumonia, bronchitis). In the registration phase III trial (MM-003), the doublet pomalidomide and dexamethasone versus high-dose dexamethasone in highly pretreated and refractory MM patients lead to significantly better PFS (median 4.0 months vs. 1 month) and OS (12.7 months vs. 8.1 months) [40]. Similarly to lenalidomide, better outcomes were seen in three drug combinations with pomalidomide, such as with bortezomib (PVD), elotuzumab (EloPD), isatuximab (IsaPD) and daratumumab (DaraPD), exceeding an mPFS of 11 months even in a highly pretreated and lenalidomide refractory population [41,42,43,44].

## 5. Summary

IMiDs have become the essential part of MM therapy. Their ability to pair with other drugs in a synergistic rather than only additive way makes them ideal partners for combined regimens with manageable toxicities [45]. The introduction of IMiDs has allowed us to gain insights into MM biology and enabled chemotherapy-free regimens with lower toxicities and better therapeutic outcomes in the majority of MM patients. With further research, we are eagerly awaiting new findings with respect to IMiD mechanisms of action as well as mechanisms of resistance in MM cells and their interaction with the tumor microenvironment.

This Special Issue is intended to focus on some aspects of new discoveries and applications of immunomodulatory drugs in basal, preclinical and clinical research.
